# Protective effects of curcumin and resveratrol on neurodegeneration and cognitive dysfunction in a streptozotocin-induced alzheimer rat model

**DOI:** 10.1038/s41598-026-48216-9

**Published:** 2026-04-18

**Authors:** Derya Öztürk Söylemez, Erdoğan Unur, Dilek Sağır

**Affiliations:** 1 Medical Services and Techniques Program, Vocational School of Health Services, Sinop University, Sinop, 57000 Turkey; 2 Department of Anatomy, Faculty of Medicine, Erciyes University, Kayseri, 38039 Turkey; 3 Department of Occupational Health and Safety, Faculty of Health Sciences, Sinop University, Sinop, 57000 Turkey

**Keywords:** Alzheimer’s disease, Curcumin, Resveratrol, Streptozotocin-induced model, Neuroprotection, Oxidative stress, Neuroinflammation, Cognitive impairment, Biochemistry, Drug discovery, Neurology, Neuroscience

## Abstract

Alzheimer’s disease (AD) is a progressive neurodegenerative disorder that is characterized by memory loss, cognitive decline, and behavioral disturbances. Given the limited efficacy of current treatments, there is an urgent need for new neuroprotective strategies. In this study, the therapeutic potential of curcumin and resveratrol was evaluated in a rat AD model induced by intracerebroventricular (ICV) administration of streptozotocin (STZ) (3 mg/kg). Subsequent to model induction, rats were administered curcumin, resveratrol, or a combination of both. The behavioral analyses, which included the Morris water maze, open field, and passive avoidance tests, revealed that both compounds significantly improved learning and memory performance. The histological and immunohistochemical findings demonstrated a decrease in caspase-3 and GFAP immunoreactivity, suggesting reduced neuronal apoptosis and astrocyte activation. The behavioral analyses, which included the Morris water maze, open field, and passive avoidance tests, revealed that both compounds significantly improved learning and memory performance. The histological and immunohistochemical findings demonstrated a decrease in caspase-3 and GFAP immunoreactivity, suggesting reduced neuronal apoptosis and astrocyte activation. Biochemical results showed an increase in total antioxidant status (TAS) and superoxide dismutase (SOD) activity, along with a decrease in total oxidative status (TOS) and TNF-α levels. Stereological evaluation also confirmed partial restoration of hippocampal volume. While curcumin and resveratrol exhibited potent neuroprotective effects when used separately, no synergistic interaction was observed when used in combination. These findings suggest that curcumin and resveratrol may serve as promising therapeutic agents for reducing oxidative stress, neuroinflammation, and neuronal degeneration associated with Alzheimer’s disease.

## Introduction

Alzheimer’s disease (AD) is a type of neurodegenerative disease that is one of the most common causes of dementia worldwide. It is estimated that approximately 44 million individuals worldwide are currently affected by AD, with projections indicating that this number is expected to rise to 115 million by the year 2050^1^. AD is a chronic progressive disorder that is characterized by behavioral and cognitive impairments. The pathological mechanisms underlying of AD are characterized by the presence of amyloid plaques, neuroinflammation, neuronal loss, and the formation of neurofibrillary tangles. One of the most salient underlying factors contributing to the development and progression of these pathologies is oxidative stress^2^. In Alzheimer’s disease, as the pathological processes progress, there is a deterioration of neural connections between the limbic system and the temporal and frontal lobes. This deterioration gives rise to a neurodegenerative picture in which the temporoparietal cortex is particularly affected, but the frontal lobe is also involved in the process. As the pathological changes progress, extensive cerebral atrophy develops^3–5^.

In recent years, research has focused on the effects of oxidative stress resulting from neuronal damage, particularly in AD, on disease progression. Rats with cognitive impairments induced by ICV administration of Stz have been proposed as a research model for understanding AD. The ICV administration of Stz has been demonstrated to induce oxidative stress and neuroinflammation^6^. At present, there is no definitive treatment for AD. Current treatment methodologies entail the administration of symptomatic treatments aimed at decelerating the progression of the disease^7^. The significance of antioxidants, vitamins, and the Mediterranean diet in the prevention of Alzheimer’s disease or the retardation of its progression is underscored^8^. In this context, plant compounds that exhibit notable natural antioxidant properties have garnered significant interest in recent years due to their therapeutic potential.

Curcumin, an active diphenolic compound, is obtained from the root of Curcuma longa (turmeric) and is used in both pharmaceuticals and as a food additive. The medication has minimal side effects and is capable of crossing the blood-brain barrier with ease^9^. A substantial number of studies have demonstrated that curcumin exhibits antioxidant, anti-allergic, anti-tumor, anti-inflammatory, anti-carcinogenic, and anti-dementia properties, in addition to free radical scavenging activity^10–13^. It has been reported that curcumin provides a certain level of neuron-protective effect against neuronal cell death caused by oxidative stress^14^. Recent studies have suggested that curcumin may have a beneficial role in neurodegenerative disorders due to its various neuroprotective properties^15,16^.

Resveratrol is a stilbene-structured polyphenol that is found naturally in many plant species, primarily red grapes, mulberries, and peanuts^17^. The compound’s antioxidant, anti-inflammatory, and anti-apoptotic properties have garnered interest in the field of neurodegenerative disease research. A substantial body of research has demonstrated that resveratrol diminishes the accumulation of amyloid plaques in animal models of Alzheimer’s disease. In addition, it has been shown to suppress oxidative stress and inflammatory responses, while promoting neuronal survival through SIRT1-mediated signaling^18,19^. These findings suggest that the compound may have a multifaceted neuroprotective potential against the pathophysiology of AD.

Recent studies have indicated that natural polyphenols may have a protective role in neurodegenerative diseases, particularly Alzheimer’s disease, in complex pathophysiological processes characterized by the interaction of oxidative stress, neuroinflammation, and apoptotic mechanisms^20,21^. Polyphenols have established neuroprotective effects by reducing oxidative stress, modulating signaling pathways such as NF-κB and Nrf2/ARE, enhancing cellular antioxidant defense systems, and protecting mitochondrial function^22,23^. Curcumin and resveratrol represent two of the most extensively studied natural compounds among these polyphenols. The two substances in question have been shown to possess notable antioxidant, anti-inflammatory, and anti-apoptotic properties. In addition, they have demonstrated promising results in the reduction of oxidative stress and neuronal cell death in the pathophysiology of Alzheimer’s disease. However, it has been reported that the combined use of these two compounds does not always produce synergistic results; in some cases, dose-dependent interactions and metabolic competition may occur^24–26^. Therefore, further research is necessary to elucidate the effects of these antioxidant compounds on the pathophysiology of Alzheimer’s disease and their potential synergistic mechanisms.

The objective of this study was to examine the impact of curcumin and resveratrol, both of which are recognized for their robust antioxidant properties, on the hippocampus—the brain region impacted in the initial stages of Alzheimer’s disease—utilizing histomorphometric, behavioral, and biochemical approaches in a rat model. The findings are expected to contribute to scientific knowledge regarding the pathophysiology of Alzheimer’s disease, a neurodegenerative disorder with increasing global prevalence, and to the development of new neuroprotective treatment strategies.

## Materials and methods

### Animals and ethical approval

In this study, 60 male Wistar albino rats (weighing 210–250 g) were obtained from Ondokuz Mayıs University Experimental Animal Application and Research Center. During the experiment, rats were kept in a controlled environment with a temperature of 22 °C and 55–60% relative humidity. Rats were kept on a 12-hour light/dark cycle and had free access to standard rat chow and tap water. The study protocol was approved by Ondokuz Mayıs University Experimental Animal Ethics Committee (Ethics Code: 2020/67). All experimental procedures were performed in accordance with the relevant guidelines and regulations. The study is reported in accordance with the ARRIVE guidelines.

### Determination of the sample size and power analysis

The total sample size was calculated using the G*Power 3.1 program. In the one-way ANOVA analysis performed, the target power was set at 0.95, and the required sample size for each group was calculated as 8 (actual power = 0.975). In order to mitigate potential risks related to animal loss and contamination during the experiment, it was determined that each group would consist of ten animals.

### Experimental design

In accordance with the protocols documented in previous studies, a single intracerebroventricular (ICV) injection of streptozotocin (STZ) (3 mg/kg in 5 µL saline) was administered on day 1 of the experiment under stereotaxic guidance to induce Alzheimer’s-like pathology. Prior to the ICV injection, the animals were anesthetized with an intraperitoneal mixture of ketamine (100 mg/kg) and xylazine (10 mg/kg) (Merck, Cat. No. K2753; X1251). They were then fixed in a stereotaxic apparatus. 5 µL of STZ (3 mg/kg) was dissolved in citrate buffer (Santa Cruz Biotechnology, Cat. No. U-9889). The solution was then drawn into a Hamilton microinjector and injected into the left lateral ventricle (0.8 mm posterior to bregma, 1.5 mm lateral to the sagittal suture, and 3.6 mm below the brain surface) at a rate of 1 µL per minute. Subsequent to the injection, the Hamilton syringe was maintained in position for a period of two minutes prior to its removal. The injection procedure was performed on the first day of the experiment, and only one injection was administered.

On the first day of the experiment, the weights of all rats were measured and recorded, and they were randomly divided into six groups: The experimental groups included Control (C), Alzheimer’s (ALZ), Alzheimer’s + Curcumin (ALZ + CUR), Alzheimer’s + Resveratrol (ALZ + RES), Alzheimer’s + Curcumin + Resveratrol (ALZ + CUR + RES), and Sham. Following the assignment of groups, only the Alzheimer’s and treatment groups received STZ injections, while the Sham group received an icv injection of serum citrate buffer, the STZ solvent. The control group was not exposed to any inducing agent.

The validation of the model was performed in parallel with studies conducted using similar protocols documented in the extant literature. Furthermore, the elevated levels of MDA and TNF-α, as well as the diminished levels of SOD, observed in the Alzheimer’s group, substantiated the effective biochemical establishment of the model^27,28^.

The treatment protocol was initiated the day after the ICV-STZ injection (day 2). In this study, the experimental group, designated as the ALZ + CUR group, received oral gavage administrations of curcumin (100 mg/kg) from Sigma-Aldrich (Cat. No: C1386) on a daily basis for a period of 21 days. In a similar manner, rats in the ALZ + RES group were administered resveratrol via oral gavage for the same duration (25 mg/kg; Merck, Cat. No: R5010). Animals in the combination group (ALZ + CUR + RES) received both agents at their individual doses for the same duration. Curcumin and resveratrol were dissolved in dimethyl sulfoxide (DMSO) immediately before administration, ensuring complete solubilization of both polyphenols; the final DMSO concentration in the administered solution was kept below 0.5% to avoid solvent-related effects. The doses of curcumin and resveratrol were selected based on the findings of previous reports that demonstrated neuroprotective activity in rodent models of Alzheimer’s disease^29,30^. These concentrations have been demonstrated to manifest antioxidant and anti-inflammatory effects without observable toxicity, and thus, they were employed as standard effective doses in the present study. The Control, ALZ, and Sham groups received the same volume of vehicle (0.5% DMSO solution) by oral gavage during the treatment period.

On the 15th day, the Open Field Test, Passive Avoidance Test, and Morris Water Maze Test were administered to the subjects in order of increasing stress levels, with at least a 24-hour rest interval between each test. In an effort to minimize the utilization of animals, all three behavioral assessments were conducted on a single cohort of subjects. Prior to the assessment, the animals were acclimated to the test chamber, and all procedures were conducted at the same time of day to prevent circadian variations. To ensure the absence of olfactory cues, each apparatus was meticulously sanitized with 70% ethanol between experimental sessions.

**Fig. 1:**
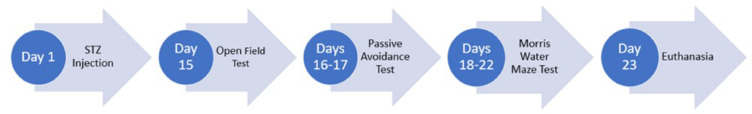
Timeline of the experimental protocol.

On the 23rd day of the experiment, the animals were weighed again and euthanized under general anesthesia with a mixture of xylazine (10 mg/kg) and ketamine (50 mg/kg). The experimental timeline, including STZ administration, behavioral assessments, and euthanasia schedule, is illustrated in Fig. 1.

Subsequent to the sacrifice of the rats, the weights of the appropriately extracted brain tissues were measured and documented. These tissues were then placed in 10% buffered formaldehyde fixative for subsequent histological analysis. The obtained blood serum samples were stored at −80 °C for subsequent biochemical analysis.

### Behavioral tests

On the 15th day, behavioral tests were initiated to evaluate the effects of treatment applications on cognitive performance. This time point is consistent with the approximately two-week period recommended in the literature for the neuroprotective effects of curcumin and resveratrol to emerge^31,32^. As indicated by earlier findings, these agents have been demonstrated to produce quantifiable behavioral improvements within a period of two weeks. The treatment process was continued until day 22 of the experiment, and during this period, the objective was to stabilize long-term antioxidant, anti-inflammatory, and anti-apoptotic responses, in addition to short-term cognitive improvement. The sacrifice was carried out on the 23rd day of the experiment. This time interval was designed to allow for the full emergence of treatment-related biochemical effects and the dissipation of acute stress responses that behavioral tests may induce.

### Open field test

The open field test is one of the tests employed to detect changes in the emotional state of animals that may arise from procedures performed on experimental animals. This approach has also been employed in the context of studying emotions that are influenced by anxiety. In the open field test employed in the study, a platform composed of white wood, subdivided into 49 equal squares on the floor, with an open top and dimensions of 100 × 100 × 30 cm (width x length x height), was utilized. Prior to the Open Field Test, all rats were acclimated to the test environment to reduce stress and locomotor changes associated with novelty. The animals were acclimated to the test chamber 24 h prior to the commencement of the experiment and permitted to navigate the environment, including the central area, for a duration of 3–5 min immediately preceding the test.

Rats were placed in the apparatus with their backs facing the open area from a specific corner and observed for five minutes while being recorded on camera. Subsequent to the use of each animal, the boxes were meticulously sanitized with 70% ethanol. The number of rearing episodes and the number of squares traversed in the periphery in the recorded videos were used to assess locomotor activity. The number of grooming episodes, the number of squares traversed in the central area, and the time spent in the central area were also used to assess the animals’ anxiety levels^33^.

### Passive avoidance test

In this experiment, a One-Trial Step-Through Passive Avoidance Set-up (Ugo-Basile model 7551, Italy) was utilized, comprising two compartments, one illuminated and one dark, separated by a guillotine door, with dimensions of 25 × 15 × 15 cm. Prior to the training period, the rats were individually placed in the light compartment and given a 30-second waiting period to acclimate to the conditions of the environment. Subsequently, all rats in the light compartment were permitted to transition into the dark compartment in sequence. Subsequent to the learning process that occurred during the habituation period, rats that were placed in the light compartment during the first phase of the test were permitted to transition into the dark compartment via the automatic opening of the guillotine door. Following their entry into the dark area, the rats were exposed to a low-dose electric shock (0.5 mA, 3 s). The latency time, defined as the duration between the onset of illumination in the light compartment and the subsequent movement of the rat into the dark compartment, was meticulously documented. The rats were subsequently returned to their cages after receiving an electric shock. Twenty-four hours after the initial learning period, the same procedure was repeated. Assuming that the rats would recall this new information, which they were thought to have learned and stored, after 24 h, the time taken to transition to the dark compartment, i.e., the retention time, was measured and recorded. However, on the second day of the experiment, the retention time of rats that did not enter the dark area within 300 s was designated as 300 s. It is noteworthy that no electric shock was administered during the experiments conducted on the second day^33^.

### Morris water maze test (MWM)

This test is widely used in the evaluation of spatial memory and learning. The experiments are conducted in a circular swimming pool with a diameter of 150 centimeters and a height of 80 centimeters. The pool contains a platform. The experimental protocol is comprised of five days, with the initial four days comprising trial phases during which learning sessions are conducted. The fifth day is designated for the probe phase, in which the memory test is administered^34^.

Before the experiments, various images were placed on the north, east and west sides of the platform in order to divide the pool imaginatively into four quadrants (southwest, southeast, northwest and northeast). The pool was filled with tap water ensuring that the platform was approximately 2 cm below the water level and the water temperature was set to 25 ± 1 °C with an automatic heater. The platform was placed in the southwest quadrant of the pool and was not moved until the end of the experiments.

During the first four days of the experiment, the subjects were released into the pool from different points. The objective of this phase was to learn the location of the platform. On each test day, subjects were released from the other three quadrants or directions where the platform was not located and allowed to swim freely for 90 s. Subjects who found the platform within the allotted time frame of 90 s were granted an additional 30 s to observe the visual cues presented. Subjects who failed to locate the platform within the allotted time (90 s) were subsequently positioned on the platform for a 30-second observation period. During this time, they were instructed to observe the visual cues and become familiar with the platform. On the fifth day, during the probe phase, the subjects’ memory was tested. To ensure a uniform color scheme, food coloring was added to the tap water in the pool to match the white color of the pool and platform. The platform was then removed. Subjects were released into the water from any of the three quadrants other than the target quadrant during the probe phase. In this phase, subjects were expected to swim directly to the location of the platform, and the time they spent in the quadrant where the platform was located (maximum 60 s) was recorded.

### Histological analyses

Brain tissues were fixed with a 10% buffered neutral formalin solution were subjected to routine histological procedures and blocked. Section 20 μm thick were taken from the paraffin blocks suitable for stereological sampling and stained with Cresyl violet (ISOLAB, Cat. No. 912.D01). Additionally, Sect. 4 μm thick were taken for immunohistochemical analysis. Neurons were classified based on their morphology and staining characteristics. Histopathological scoring was performed by evaluating the entire CA region of the hippocampus, and the final score was assigned based on the overall neuronal damage observed in the pyramidal cell layer. The photomicrographs presented in the figures represent representative areas from the CA2–CA3 subfields.

A semi-quantitative scoring system ranging from 0 to 3 was used to evaluate hippocampal sections stained with cresyl violet^35^. Histopathological scoring was performed by examining the pyramidal neuronal layer of the CA region of the hippocampus. The evaluation was conducted independently by two blinded observers, and the final score for each section was determined by consensus. For each section, three randomly selected microscopic fields were evaluated at the same magnification, and the average score was calculated for each animal. The scoring was based on the morphological characteristics of neurons and the degree of neuronal degeneration observed.

Score 0 : Normal. The neurons are round or oval with light-colored nuclei and distinct Nissl bodies. The cells appear healthy.

Score 1: Mild damage. Damage is observed in a small number of neurons. The cytoplasms appear pale, or the nuclei are slightly pyknotic.

Score 2: Moderate damage. Neurons show significant damage. Cytoplasms are reduced, and nuclei are hyperchromatic and disrupted.

Score 3: Severe damage. Neurons are largely lost. Cell structure becomes unrecognizable. There are signs of severe damage, such as gliosis and vacuolization.

### GFAP immunocytochemistry

The sections were placed in an oven at 50 °C overnight for deparaffinization, then clarified in xylene and rehydrated in alcohol series. To elicit antigen, sections were heated in 10 mM citrated buffer (pH: 6) in a 400-watt microwave oven for 20 min and then allowed to cool in the same buffer for 30 min at room temperature. For the elimination of endogenous peroxidase activity in the tissues, the sections were treated with a 3% hydrogen peroxide solution and left for 15 min. Then, all staining steps were performed using a fully automated immunohistochemistry instrument (VENTANA, Benchmark/I.T., Ventana Medical Systems, USA) under constant temperature and conditions. GFAP monoclonal antibody (Abcam, Cat. No. ab33922, RRID: AB_732571) was used as the primary antibody to make the targeted protein visible in autostained sections. The incubation time with antibodies was set at 2 h. After the staining process was completed, the sections were passed through alcohol series (70%, 80%, 96% and 99% ethyl alcohol) for 2 min each. The sections were dried at room temperature, kept in xylol for 15 min and covered with entellan. For the evaluation of immunohistochemical staining, all sections were examined under a light microscope.

The evaluation of GFAP staining results was conducted by means of a random selection of 10 different areas at 20x magnification for each animal. The analysis encompassed five sections and 50 images for each animal. A total of 200 cells were counted in each field, with the cells being classified as either (+) or (−). The density of (+) cells in each animal was then calculated using the following formula^36^.

$$GFAP\,(+)\,cell\,density=\frac{GFAP(+)\,stained\,cell\,number}{Total\,number\,of\,GFAP(+)\,and\,GFAP\,(-)\,stained\,cells}\times 100$$

### Caspase-3 immunocytochemistry

5 μm thick sections from brain tissues were automatically stained for caspase-3 immunohistochemical reactivity using the VENTANA BenchMark GX System (Ventana Medical Systems, Inc.). Antigenic marker regions for caspase-3 were unfolded with vapor in citrate buffer for 60 min. For caspase-3 staining, IgG grade Caspase-3 antibody (Santa Cruz, Cat. No. sc-65497, RRID: AB_2054782) and mouse monoclonal IgG2a (kappa light chain) were diluted 1:80. The sections were incubated in antibody solution for 32 min at 37 °C, followed by the use of the ultraView Universal DAB imaging kit (Ventana Medical Systems, Inc.). DAB (Sigma-Aldrich, Cat. No. D8001) was used as chromogen and staining was counterstained with hematoxylin. The specificity of the staining was confirmed by processing negative control sections in which the primary antibody was not used.

The number of Caspase-3 (+) cells was counted and divided by the overall cell count for each field. Each section was evaluated on average across six fields. The percentage of stained cells was assessed: 0 for no stained cells; 1 for 25%−50% stained cells; 2 for 25%−50% stained cells; 3 for > 50% stained cells. Staining intensity was scored as follows: none (0); low (1); moderate (2); severe (3). The Immunostaining Intensity Distribution Index (IIDI) was calculated using the formula: IIDI = stained cells x staining density. The IIDI represents the average of all six fields^37^.

### Stereological analysis

Considering previous studies, the sampling interval of the hippocampal volume in the brain was accepted as 1/6 (Systematic). According to this sampling interval, 20 μm-thick sections were taken from the brain blocks and 1/6 systematic and uniform random sampling was performed. The Cavalieri method was used to measure the area of the sections and to calculate the total volume of the hippocampus. A Stereo-Investigator (Microbrightfield, Colchester, VT) was used in the study. This device had special software that included a dotted area ruler for area calculation with the Cavalieri method. The total volume was automatically calculated by the software according to the Cavalieri principle^37^.

### Biochemical analysis

#### Collection of serum samples

The blood obtained from the heart under anesthesia was transferred into serum tubes (BD SST™ II Advance Serum Separator Tube). After the blood was collected in serum tubes, it was gently turned upside down 5–6 times and allowed to clot for 30 min. Following the clotting, the serum tubes were centrifuged at 1300–2000 x g for 10 min at room temperature (25 °C). After centrifugation, the sera were collected in 0.5 ml microcentrifuge tubes. They were then placed in a deep freezer at −80 ˚C and stored until the day of measurement.

#### Determination of total antioxidant and oxidant levels (TAS/TOS)

Total antioxidant status (TAS) and total oxidant status (TOS) levels in serum were determined using commercial colorimetric assay kits (Bioassay Technology Laboratory, Shanghai, China; TAS: E1710Ra; TOS: E1512Ra). Measurements were performed according to the manufacturer’s instructions. The analysis is based on measuring the color intensity formed at the end of the reaction at a wavelength of 450 nm using a microplate reader. The results were calculated as follows: mmol Trolox equivalent/L for TAS and µmol H₂O₂ equivalent/L for TOS. All samples were tested in duplicate.

#### Serum MDA, SOD, TNF-α, and caspase-3 ELISA assays

The levels of oxidative damage, antioxidant enzyme activity, inflammation, and apoptosis markers in rat serum samples were analyzed using commercial ELISA kits. Malondialdehyde (MDA), superoxide dismutase (SOD), tumor necrosis factor-alpha (TNF-α), and caspase-3 levels were determined using kits from BT-Laboratory (Shanghai, China) (MDA: E0156Ra; SOD: E0168Ra; TNF-α: E0764Ra; caspase-3: E1648Ra).

The double-antibody sandwich ELISA principle, as defined by the manufacturer, was applied in all kits. ELISA plates were provided pre-coated with a capture antibody that was specific to the target protein. Serum samples stored at − 80 °C were thawed prior to analysis, and 50 µl of sample was transferred to each well. Subsequent to the completion of the incubation period, unbound material was removed through the implementation of appropriate washing steps.

Subsequently, the biotin-conjugated detection antibody specific for the target protein provided in the kit was added, thereby forming the sandwich structure. Subsequent to the binding of the detection antibody, the streptavidin-horseradish peroxidase (HRP) conjugate was applied, thereby forming an enzyme-linked complex via the biotin–streptavidin interaction. The addition of TMB substrate at 37 °C was initiated to facilitate the desired color development, and the reaction was halted with the application of a stop solution following the completion of the designated incubation period. Absorbance values were measured at a wavelength of 450 nanometers (nm).

The concentration calculations were performed using standard curves provided by the manufacturer, and all samples were tested in duplicate to ensure reliability. As the technical documentation provided by the manufacturer fails to offer clone numbers or species information for capture and detection antibodies, the method section presents antibody details using kit codes. These kit codes are based on the information provided by the manufacturer.

#### Randomization and blinding procedures

In this study, all experimental procedures were conducted in accordance with randomization principles. The allocation of group codes to participants was conducted by a researcher who was not affiliated with the experimental team. Behavioral tests, biochemical measurements, histopathological scoring, immunohistochemical evaluations, and stereological volume analyses were performed under conditions of complete blinding. The researchers who conducted these analyses were kept unaware of the group distributions until the statistical evaluation of the study was completed. Consequently, observer bias was mitigated, and the integrity of the data was maintained.

#### Data collection and exclusion criteria

During the experiment, no mortalities or instances of behavioral exclusion were observed. Throughout the study period, the subjects exhibited robust health, successfully completing the behavioral assessments and treatment procedures. All data obtained were included in the final statistical analysis.

#### Statistical analysis

The numerical data of the groups obtained in the study were statistically evaluated using the SPSS program (SPSS version 21.0; SPSS Inc., Chicago, IL, USA). The data used were expressed as mean±standard deviation. As a result of the normality and homogeneity tests of the data of the groups, the differences between the groups with normal distribution were evaluated through One-Way ANOVA and Tukey tests, while the groups without normal distribution were analyzed through Kruskal-Wallis and Tamhane’s T2 tests. A value of *p* < 0.05 was considered statistically significant in statistical evaluation. (*) indicates that *p* < 0.05, (**) indicates that *p* < 0.01, (***) indicates that *p* < 0.001.

## Results

### Behavioral test findings

#### Open field test

**Fig. 2:**
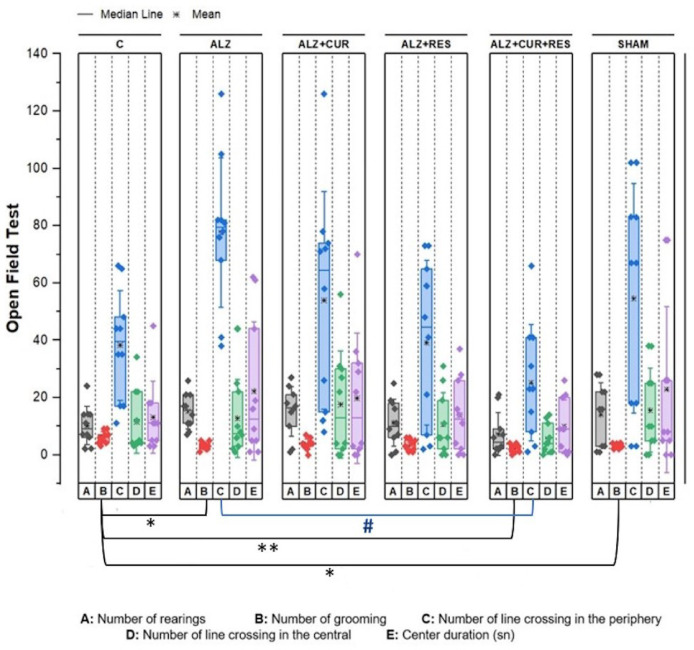
Effects of curcumin and resveratrol on anxiety-related behaviors in Alzheimer model rats during the open field test. ^*^ *p* < 0.05, ^**^ *p* < 0.01, ^***^ *p* < 0.001 vs. Control group; ^#^*p* < 0.05 vs. ALZ group.

The results of the Open Field Test are presented in Fig. 2. A comparative analysis of the number of rearing episodes, a parameter of the open field test, revealed no statistically significant differences between the groups (*p* > 0.05). When the number of grooming episodes was evaluated, the Control group exhibited a significantly higher number of grooming episodes in comparison to the ALZ (*p* < 0.05), ALZ + CUR+RES (*p* < 0.01), and SHAM groups (*p* < 0.05). The number of line crossings on the 24 squares adjacent to the walls located on the periphery of the open field platform was significantly higher in the ALZ group than in the ALZ + CUR+RES group (*p* < 0.01). No statistically significant differences were observed between the study groups in terms of the parameters of center line count and center dwell time, which represent the time spent on these squares (*p* > 0.05).

#### Passive avoidance test

**Fig. 3:**
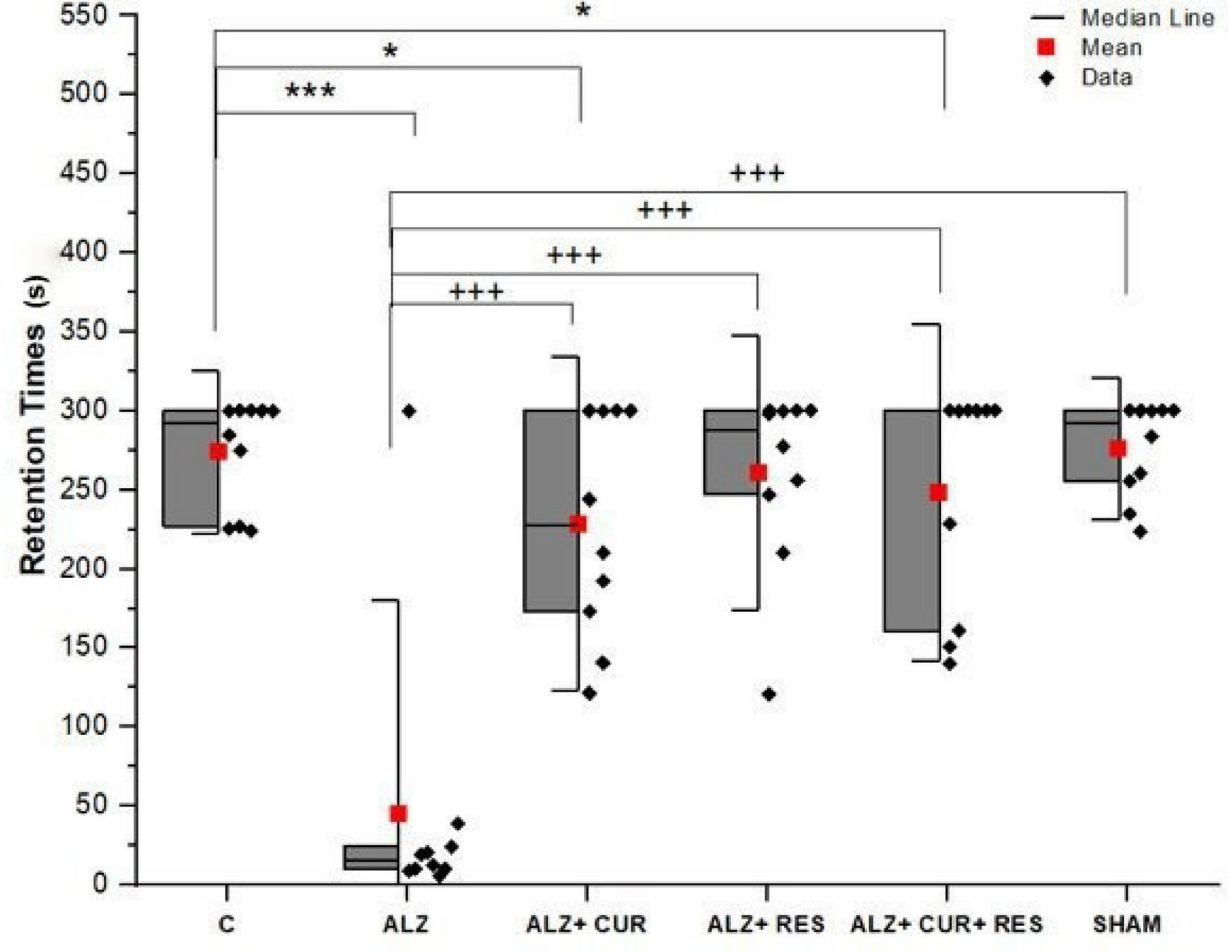
Retention times of rats in all groups in the passive avoidance test. ^*^ *p* < 0.05, ^***^ *p* < 0.001 vs. Control group; ^###^*p* < 0.001 vs. ALZ group.

In the passive avoidance test, a widely utilized tool for evaluating short- and long-term memory, the mean values and standard errors of the times (s) required for the groups to enter the dark zone are illustrated in Fig. 3. According to statistical analysis, the average time taken by the ALZ group to enter the dark zone was 45.08 ± 28.49 s, which was significantly lower than that of all other groups (*p* < 0.001). A subsequent comparison of the times taken to enter the dark zone between groups revealed no significant difference between the Control, SHAM, and ALZ + RES groups. In the ALZ + CUR+RES and ALZ + CUR groups, the transition time to the dark zone was significantly lower than in the Control, SHAM, and ALZ + RES groups, but higher than in the ALZ group (*p* < 0.001).

Rats that did not enter the dark compartment within 300 s were assigned a maximum retention time of 300 s, in accordance with the experimental protocol. Overlapping data points at the upper limit represent animals that exhibited complete avoidance behavior.

#### Morris water maze test

**Fig. 4:**
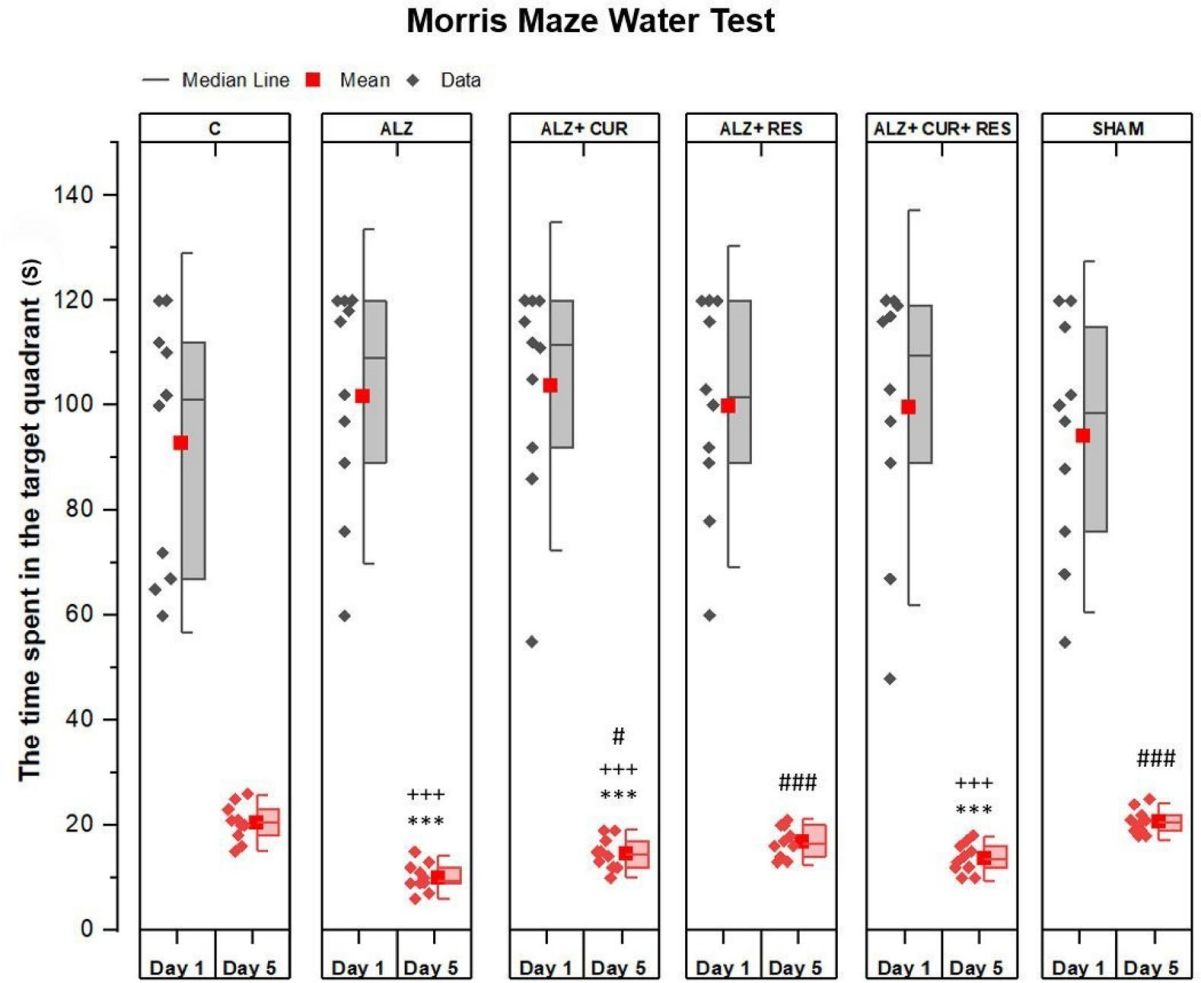
Effects of curcumin and resveratrol on spatial learning and memory in Alzheimer’s model rats in the Morris water maze (MWM). ^***^ *p* < 0.001 vs. Control group; ^#^ *p* < 0.05, ^###^ *p* < 0.001 vs. ALZ group; ^+++^ *p* < 0.001 vs. ALZ + RES group.

The mean values for the duration of the search for the platform and the time spent in the quadrant where the platform was located on the test day are presented in Fig. 4. In the intergroup comparisons, no significant difference was found between the groups on the first day of the test phase (*p* > 0.05). When analyzing the probe phase times on the 5th day of the Morris water maze test, it was observed that the Control group spent more time in the target quadrant compared to the ALZ, ALZ + CUR, and ALZ + CUR+RES groups, and a statistically significant difference was observed (*p* < 0.001). The time spent in the target quadrant by the ALZ group was significantly lower than that of the ALZ + CUR (*p* < 0.05), ALZ + RES (*p* < 0.001), and SHAM (*p* < 0.001) groups. The time spent in the target quadrant by rats in the ALZ + RES group was similar to that in the Control and SHAM groups (*p* > 0.05), but significantly higher than that in the ALZ + CUR and ALZ + CUR+RES groups (*p* < 0.001). The ALZ group exhibited the shortest duration of time spent in the target quadrant, with an average of 10.10 ± 0.86 s.

### Stereological findings

#### Total hippocampal volume

**Fig. 5:**
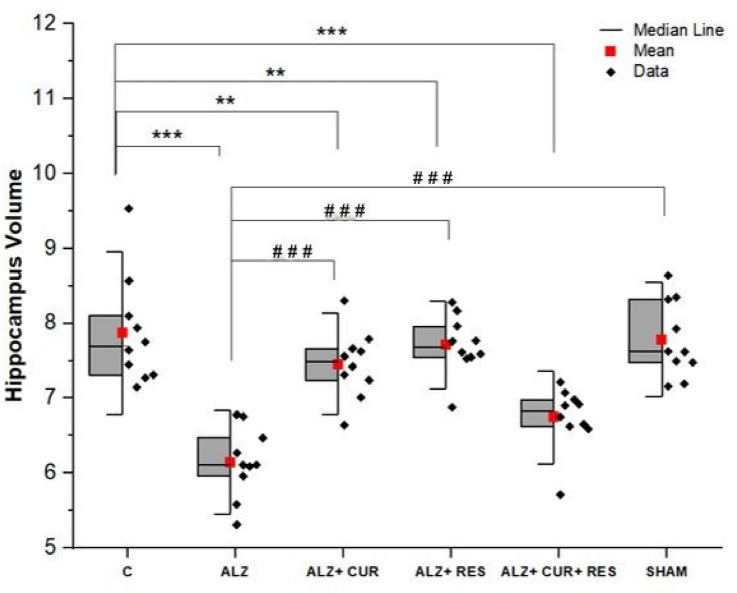
Hippocampal volumes of rats in all groups. ^**^ *p* < 0.01, ^***^ *p* < 0.001 vs. Control group; ^###^ *p* < 0.001 vs. ALZ group.

The volumetric analysis of the hippocampus was conducted using stereological methods, as previously outlined (Fig. 5). Subsequent to statistical evaluation of the obtained data, the lowest area volume was observed in the ALZ group. The total hippocampal volume of the ALZ group was significantly lower than that of the Control, ALZ + CUR, ALZ + RES, and SHAM groups (*p* < 0.001). The hippocampal volumes of the ALZ + CUR and ALZ + RES groups were statistically significantly lower than that of the Control group. The total hippocampal volume of the ALZ + CUR+RES group was significantly lower than that of the Control, ALZ + CUR, ALZ + RES, and SHAM groups (*p* < 0.001). Conversely, no statistically significant difference was observed in the mean hippocampal volumes of the ALZ and ALZ + CUR+RES groups (*p* > 0.05).

### Histopathologic findings

#### Cresyl violet stain

**Fig. 6:**
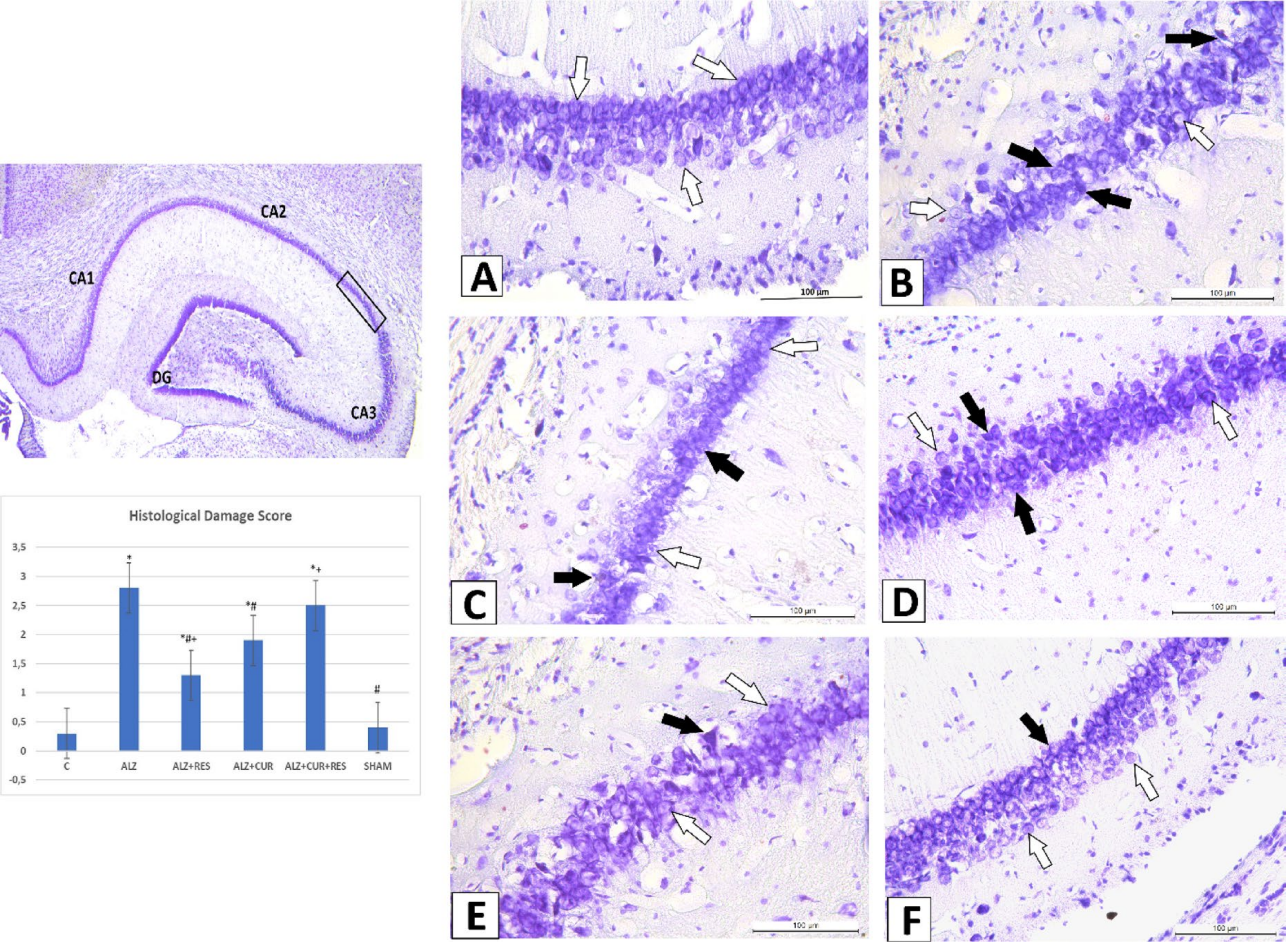
Representative photomicrographs of hippocampal sections from all experimental groups. (**A**) Low-magnification overview of the hippocampus showing the sampled area from which the high-magnification images were obtained. High-magnification photomicrographs were captured from the same anatomical level and comparable regions of the CA pyramidal layer at identical magnification to ensure comparability between groups. White arrows indicate morphologically intact neurons, whereas black arrows indicate degenerated neurons (**B**: Control; **C**: ALZ; **D**: ALZ + CUR; **E**: ALZ + RES; **F**: ALZ + CUR+RES; **G**: SHAM). Staining: Cresyl violet; magnification ×400. (H) Semi-quantitative histological damage scores (HDS) calculated from three randomly selected microscopic fields within the CA region of the hippocampus in each section, averaged for each animal. ^*^ *p* < 0.05 vs. Control group; ^#^*p* < 0.05 vs. ALZ group; ^+^*p* < 0.05 vs. ALZ + RES group.

The histopathological damage scores in the hippocampal sections of rats in all groups are presented in the graph on the right side of Fig. 6G. Following comprehensive analysis, no significant abnormalities were detected in the general structure of the hippocampus and neurons in the control and SHAM groups, and cell boundaries remained clearly defined. A detailed analysis of the general structure of the hippocampal region in brain sections from the ALZ group revealed the presence of neurons characterized by euchromatic nuclei and narrow, dark cytoplasm. The cell boundaries were indistinct in the majority of neurons within this group, and a substantial loss of cells was documented. Notably, the boundaries of neurons undergoing degeneration remained indistinguishable. Furthermore, the size of neurons that retained their structural integrity was also reduced. Furthermore, degenerative structures containing potential cell remnants were frequently observed. In the ALZ + CUR and ALZ + RES groups, it was evident that the degeneration and cell loss observed in the ALZ group were less pronounced, and the number of neurons with normal structure was higher. In the ALZ + CUR+RES group, the histological appearance of the hippocampus exhibited notable similarity to that of the ALZ group. In this group, the density of degenerative cells exhibiting reduced nucleus size and narrow, darkly stained cytoplasm was significantly higher than in the control and SHAM groups. Moreover, in comparison with the ALZ + CUR and ALZ + RES groups, the density of healthy neurons was reduced, while the density of degenerative neurons was elevated (Fig. 6A-G).

### Immunohistochemical findings

#### GFAP ımmunohistochemistry

**Fig. 7:**
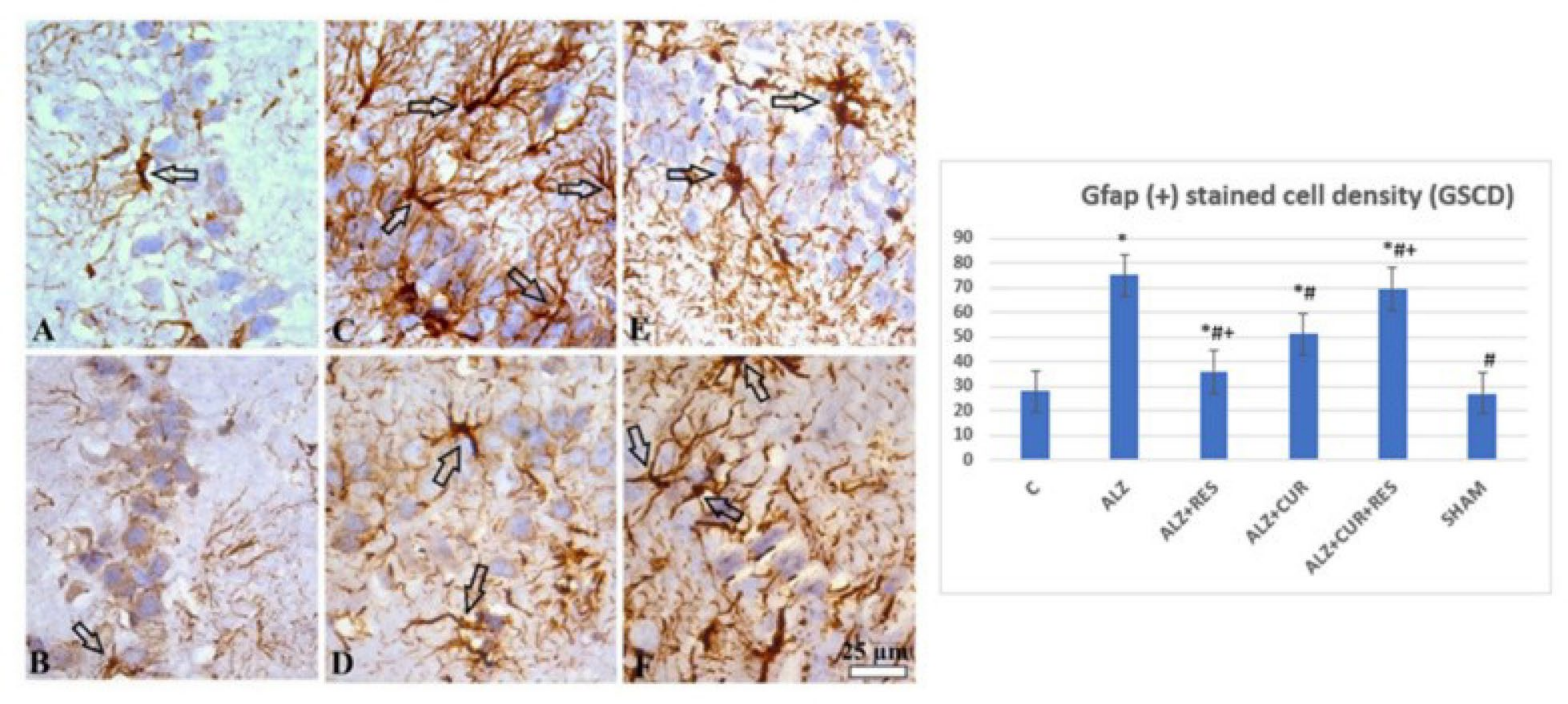
**Left panel**: Representative photomicrographs showing the distribution of GFAP-positive (GFAP⁺) astrocytes in the CA region of the hippocampus. Arrows indicate GFAP⁺ cells. Panels correspond to experimental groups as follows: (A) Control, (B) SHAM, (C) ALZ, (D) ALZ + RES, (E) ALZ + CUR, and (F) ALZ + CUR+RES. Magnification: ×1000. **Right panel**: Quantitative analysis of GFAP-positive cell density (GSCD). ^*^p < 0.05 vs. Control group; ^#^*p* < 0.05 vs. ALZ group; ^+^*p* < 0.05 vs. ALZ + RES group.

The density of GFAP-positive cells was evaluated across the hippocampal regions of all groups (Fig. 7). A marked increase in GFAP (+) cell density was detected in the ALZ group compared with all other groups (*p* < 0.05). These cells also displayed pronounced cytoplasmic hypertrophy and more extensively branched processes. No significant difference was found between the Control and SHAM groups (*p* > 0.05). The ALZ + CUR group showed a mild increase in GFAP (+) cell density relative to the Control group, whereas the ALZ + CUR+RES group exhibited a GFAP (+) profile comparable to that of the ALZ group (*p* > 0.05). Both ALZ + CUR and ALZ + RES groups demonstrated higher GFAP (+) cell numbers compared with the Control group (*p* < 0.05), although this increase was substantially less pronounced than that observed in the ALZ group. Furthermore, GFAP-positive astrocytes in the ALZ + CUR and ALZ + RES groups displayed noticeably reduced cytoplasmic process length and branching compared with the ALZ group. Notably, the cellular morphology and GFAP (+) density observed in the ALZ + RES group were more similar to those of the Control group.

### Caspase-3 ımmunohistochemistry

**Fig. 8:**
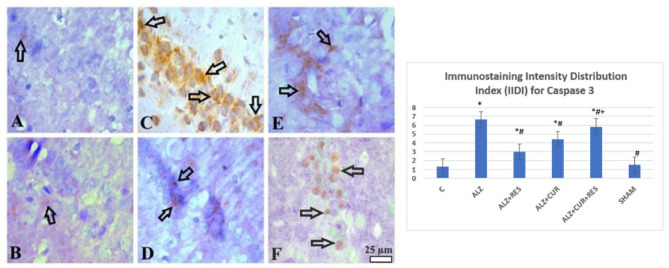
Left panel: Light microscopic images demonstrating caspase-3 immunoreactivity in hippocampal sections from all experimental groups. Arrows indicate caspase-3–positive cells. Panels correspond to the following groups: (A) Control, (B) SHAM, (C) ALZ, (D) ALZ + RES, (E) ALZ + CUR, and (F) ALZ + CUR+RES. Magnification: ×1000. Right panel: Immunostaining Intensity Distribution Index (IIDI) values for caspase-3 immunoreactivity across groups. *p < 0.05 vs. Control group; #*p* < 0.05 vs. ALZ group; +*p* < 0.05 vs. ALZ + RES group.

Immunohistochemical analysis was performed using caspase-3, and brain sections from the groups were examined. The Immunohistochemical Staining Intensity Distribution Index (IIDI) was subsequently calculated (Fig. 8). The staining intensity in the cytoplasm of the hippocampal cells exhibited variability from cell to cell. The presence of high levels of apoptosis was indicated by dark brown staining, while no staining was observed in areas showing weak activity. The groups were evaluated based on the degree of dark staining. In this context, no significant difference was observed between the control and SHAM groups (*p* > 0.05). The IIDI value was found to be significantly higher in the ALZ group compared to the Control group (*p* < 0.05). The study revealed no statistically significant difference between the ALZ + CUR+RES and ALZ groups (*p* > 0.05). While the IIDI value of the ALZ + CUR and ALZ + RES groups exceeded that of the Control group, it was notably lower than that of the ALZ group (*p* < 0.05).

### Biochemical findings

#### Changes in serum oxidative and antioxidant parameters (TAS, TOS, MDA, SOD)

The mean ± standard deviation values for serum TAS, TOS, MDA, and SOD levels are detailed in Table 1. Serum TAS levels, which were highest in the control group, were significantly reduced in the ALZ group (*p* < 0.05). A similar trend was observed in the ALZ + CUR+RES group, which exhibited a significant decrease in comparison to the control group (*p* < 0.05). In contrast, TAS levels in the ALZ + RES and SHAM groups exhibited a marked increase compared to the ALZ group (*p* < 0.05), indicating that resveratrol, when administered alone, led to a partial enhancement in antioxidant capacity.

Serum TOS levels revealed a significant increase in oxidative load in the ALZ, ALZ + CUR, ALZ + RES, and ALZ + CUR+RES groups compared with the Control group (*p* < 0.05). The elevation in TOS observed in the ALZ group was greater than that of the ALZ + CUR and ALZ + RES groups (*p* < 0.05), indicating that both curcumin and resveratrol exerted a partial ameliorative effect on oxidative burden. However, no statistically significant difference was detected between the ALZ and ALZ + CUR+RES groups (*p* > 0.05). These findings suggest that the oxidative load-reducing efficacy of the combined treatment is more limited compared with the individual therapies.

Serum MDA analysis demonstrated that the ALZ group exhibited markedly elevated MDA levels compared with the ALZ + CUR and ALZ + RES groups (*p* < 0.001). No significant difference was detected between the ALZ and ALZ + CUR+RES groups (*p* > 0.05). As expected, the Control group showed substantially lower MDA levels than all treatment groups, while no difference was observed between the Control and SHAM groups (*p* > 0.05). Among the treatment groups, the ALZ + RES group displayed the lowest mean MDA values, which were significantly reduced compared with both the ALZ + CUR and ALZ + CUR+RES groups (*p* < 0.001). Although MDA levels in the ALZ + CUR group were lower than in the ALZ + CUR+RES group (*p* < 0.001), they remained significantly higher than those of the SHAM group (*p* < 0.01).

Evaluation of serum SOD levels revealed a pronounced suppression of antioxidant enzyme activity in the ALZ group. SOD concentrations in the ALZ group were significantly lower than those observed in both the ALZ + CUR and ALZ + RES groups (*p* < 0.001). Conversely, no statistically significant difference was detected between the ALZ and ALZ + CUR+RES groups (*p* > 0.05). The Control group exhibited the highest SOD levels among all groups, with no difference between the Control and SHAM groups (*p* > 0.05). When the treatment groups were compared, the ALZ + RES group displayed markedly higher SOD activity than both the ALZ + CUR and ALZ + CUR+RES groups (*p* < 0.001). Although SOD levels in the ALZ + CUR group were higher than those in the combination group (*p* < 0.01), they remained significantly lower than those of the SHAM group (*p* < 0.01).

#### Alterations in serum ınflammatory and apoptotic markers (TNF-α and Caspase-3)

Analysis of serum TNF-α levels revealed a pronounced elevation in the ALZ group. TNF-α concentrations in this group were significantly higher than those of the ALZ + CUR and ALZ + RES groups (*p* < 0.001). In contrast, no statistically significant difference was observed between the ALZ and ALZ + CUR+RES groups (*p* > 0.05). The Control group exhibited markedly lower TNF-α levels than all treatment groups (*p* < 0.001), and no difference was detected between the Control and SHAM groups (*p* > 0.05). Comparison of the treatment groups showed that the ALZ + RES group had considerably lower TNF-α levels than both the ALZ + CUR and ALZ + CUR+RES groups (*p* < 0.001). Although TNF-α concentrations in the ALZ + CUR group were lower than those in the combination group (*p* < 0.001), they remained significantly elevated compared with the SHAM group (*p* < 0.01).

Serum Caspase-3 analysis revealed a robust activation of apoptotic pathways in the ALZ group, which exhibited significantly higher Caspase-3 levels than the ALZ + CUR and ALZ + RES groups (*p* < 0.001). The ALZ + CUR+RES group, however, presented Caspase-3 concentrations comparable to those of the ALZ group (*p* > 0.05), suggesting that the combined therapy failed to exert additional anti-apoptotic effects. Caspase-3 values were lowest in the Control group, and were statistically indistinguishable from those of the SHAM animals (*p* > 0.05). Among the treatment arms, the ALZ + RES group showed the greatest attenuation of Caspase-3, with significantly lower levels than both the ALZ + CUR and ALZ + CUR+RES groups (*p* < 0.001). Although Caspase-3 concentrations in the ALZ + CUR group were reduced compared with the combination group (*p* < 0.01), they still exceeded those observed in the SHAM group (*p* < 0.01).

**Table 1:** Serum TAS, TOS, MDA, SOD, TNF-α, and caspase-3 levels in all experimental groups (Mean ± SD).

	C	ALZ	ALZ + CUR	ALZ + RES	ALZ + CUR+RES	SHAM
TAS (mmol Trolox equivalent/L)	1,178 ± 0,322	0,698 ± 0,316^*^	0,960 ± 0,248	1,198 ± 0,234^#^	0,794 ± 0,442^*^	1,068 ± 0,310^#^
TOS (µmol H₂O₂ equivalent/L)	1,032 ± 0,292	2,381 ± 0,599^***^	1,706 ± 0,430^*^	1,568 ± 0,300^*#^	2,186 ± 0,685^**^	1,148 ± 0,244^##^
MDA (nmol/ml)	1,009 ± 0,157	4,195 ± 0,227^***^	2,835 ± 0,293 ^***###+++^	2,269 ± 0,218 ^***###^	4,084 ± 0,248 ^***+++!!!^	1,033 ± 0,174 ^###!!^
SOD (U/mL)	114,792 ± 4,126	72,502 ± 5,027^***^	87,187 ± 2,713 ^***###+++^	97,107 ± 2,195 ^***###^	76,449 ± 3,955 ^***+++!!^	115,020 ± 4,159^###!!^
TNF-α (pg/ML)	31,108 ± 0,786	100,233 ± 2,133^***^	70,710 ± 3,607 ^***##+++^	64,050 ± 5,985 ^***###^	99,811 ± 2,582 ^***+++!!!^	31,372 ± 1,121 ^###!!^
Caspase-3 (ng/mL)	1,923 ± 0,034	6,440 ± 0,231^***^	4,571 ± 0,207 ^***###+++^	3,464 ± 0,163 ^***###^	6,352 ± 0,310 ^***+++!!^	1,884 ± 0,054 ^###!!!^

^*^ *p* < 0.05, ^**^ *p* < 0.01, ^***^ *p* < 0.001 vs. Control group; ^#^ *p* < 0.05, ^##^ *p* < 0.01, ^###^ *p* < 0.001 vs. ALZ group; ^+^*p* < 0.05, ^++^ *p* < 0.01, ^+++^ *p* < 0.001 vs. ALZ + RES group; ^!^ *p* < 0.05, ^!!^ *p* < 0.01, ^!!!^ *p* < 0.001 vs. ALZ + CUR group.

## Discussion

Alzheimer’s disease has emerged as a primary area of focus in scientific research due to its escalating prevalence as a public health concern within aging populations^38–40^. This neurodegenerative disorder, which profoundly impairs cognitive functions and overall quality of life, highlights the urgent global need for effective therapeutic and preventive strategies^41,42^. In accordance with this requirement, the objective of our study was to examine the possible neuroprotective effects of curcumin and resveratrol on the hippocampus in an Alzheimer’s disease model. In this context, the potential therapeutic contributions of these compounds to the disease process were evaluated using a comprehensive approach involving histomorphometric, behavioral, and biochemical analyses.

In the open field test, a widely utilized method for evaluating anxiety-like behaviors and locomotor activity in animals, it has been established that a reduction in the time spent in the central area is indicative of heightened anxiety levels^43,44^.. Moreover, increased locomotor activity has been reported in the literature to parallel heightened anxiety levels, whereas elevated grooming behavior is conside^45^.

Two noteworthy findings were obtained during the open field test in this study. Initially, a marked augmentation in grooming behavior was evident in the control group in comparison to the other groups. Secondly, a marked increase in the number of line crossings in the ALZ group was observed. The results of this study indicate that the heightened locomotor activity and marked tigmotaxis observed, particularly in the ALZ group, may be associated with elevated anxiety levels.

In the present study, learning and memory functions were evaluated using the Passive Avoidance and Morris Water Maze tests in STZ-treated rats. Both assessments revealed a pronounced impairment in cognitive performance in the ALZ group. In the Passive Avoidance Test, rats in the ALZ group exhibited a markedly shortened latency to enter the dark compartment, indicating a substantial deficit in aversive memory. In contrast, curcumin and resveratrol treatments alleviated these impairments to varying degrees. During the final day of the Morris Water Maze task, the ALZ group demonstrated a significant reduction in the time spent in the target quadrant, further confirming spatial memory deterioration. Notably, the resveratrol-treated group displayed performance levels closely approaching those of the Control group and outperformed both the ALZ + CUR and ALZ + CUR+RES groups.

Collectively, these findings suggest that resveratrol exerts a more robust protective effect on cognitive function than curcumin under STZ-induced neurodegenerative conditionsCognitive impairments induced by STZ have been strongly linked to increased oxidative stress and heightened inflammatory responses within the brain. These alterations contribute to neuronal injury, disruption of synaptic integrity, and subsequent deficits in memory processing. In addition, STZ is known to exert cytotoxic effects on pancreatic β-cells, leading to reduced insulin production and the development of insulin resistance. Such disturbances in glucose metabolism further compromise cerebral energy homeostasis, thereby exacerbating the decline in cognitive function^46,47^.

In the present study, the total antioxidant status (TAS), total oxidant status (TOS), malondialdehyde (MDA), and superoxide dismutase (SOD) levels were analyzed to evaluate systemic oxidative balance. In the ALZ group, a significant increase in TOS and MDA levels and a significant decrease in TAS and SOD levels were observed. These results suggest that STZ weakens cellular antioxidant defense mechanisms by increasing the production of reactive oxygen species. In the groups treated with curcumin and resveratrol, a significant decrease in TOS and MDA levels and a marked increase in SOD activity were observed. These findings suggest that both compounds may have the capacity to preserve neuronal integrity by mitigating oxidative stress-induced damage.

Furthermore, TNF-α levels were found to be significantly elevated in the ALZ group, and this increase was markedly reduced by curcumin and resveratrol administration. This outcome lends further credence to the hypothesis that the suppression of proinflammatory cytokine production by both compounds contributes to a weakened inflammatory response. Concurrently, serum caspase-3 levels were observed to be elevated in the ALZ group, and this augmentation was markedly diminished with resveratrol and curcumin administration.

Consistent with previous findings, several studies have demonstrated that curcumin and resveratrol mitigate oxidative stress by scavenging free radicals and suppressing inflammatory cytokine production, effects attributed to their potent antioxidant properties^48,49^. Curcumin has been reported to enhance synaptic plasticity by increasing BDNF levels, thereby improving learning and memory performance and supporting hippocampal neuronal integrity^50,51^. Similarly, resveratrol has been reported to enhance energy metabolism by preserving mitochondrial function, attenuating oxidative stress responses, and promoting neuronal survival^23^. Moreover, previous studies have shown that polyphenols can inhibit the formation of toxic Aβ oligomers and protofibrillar intermediates, thereby slowing the progression of neurodegenerative processes^52^.

The neuroprotective effects of curcumin and resveratrol in STZ-based Alzheimer’s models have also been synthesized in a recent systematic review of animal studies^53,54^. The mechanistic insights and behavioral improvements reported in that review are consistent with the findings obtained in the present study.

In the present study, GFAP and caspase-3 immunohistochemical markers were also employed to evaluate neuronal injury at the cellular level. GFAP immunoreactivity demonstrated a marked increase in astrocytic activation in the ALZ group. The pronounced GFAP positivity observed particularly within the hippocampal and periventricular regions indicates a region-specific escalation of neuroinflammatory activity. This astrocytic response can be interpreted as part of the pathogenic cascade associated with the established neurotoxic mechanisms of STZ.

STZ administration is known to induce DNA damage in neurons, which subsequently leads to the activation of poly (ADP-ribose) polymerase (PARP)^55^. Excessive PARP activation depletes intracellular NAD⁺ and ATP reserves, thereby disrupting cellular energy homeostasis. This energetic collapse increases mitochondrial membrane permeability, promoting the release of cytochrome c into the cytosol and triggering the activation of caspase-9 and caspase-3^56,57^. In addition, STZ has been shown to exacerbate mitochondrial dysfunction by elevating the production of reactive oxygen species (ROS) and to potentiate apoptotic signaling through the upregulation of pro-inflammatory cytokines such as TNF-α and IL-1β^58^. In the present study, the neuroprotective actions of curcumin and resveratrol were clearly evident against the pathological mechanisms induced by STZ. Both compounds markedly attenuated STZ-associated oxidative stress and inflammatory responses. Notably, resveratrol appeared to exert a more pronounced suppressive effect on astrocytic activation, likely through its potent antioxidant and anti-inflammatory properties. The reduction of oxidative burden in astrocytes, together with the suppression of inflammation and apoptosis, is considered a critical therapeutic strategy for mitigating neurodegenerative progression. Consistent with these observations, previous in vivo and in vitro studies have similarly reported that resveratrol and curcumin alleviate astrocytic activation by reducing oxidative stress and dampening pro-inflammatory signaling^59,60^.

In line with the astrocytic response, immunohistochemical evaluation of caspase-3 demonstrated parallel alterations in apoptotic activity. The density of caspase-3–positive cells in the hippocampus was markedly elevated in the ALZ group, whereas curcumin- and resveratrol-treated groups exhibited significant reductions in apoptotic labeling. Previous studies have reported that curcumin mitigates inflammation and oxidative stress, enhances the expression of anti-apoptotic proteins such as Bcl-2, and suppresses caspase-3 activation^61,62^. Similarly, resveratrol has been shown to preserve mitochondrial function, downregulate the expression of pro-apoptotic proteins, and promote neuronal survival^63–65^. The observations obtained in the present study align well with these proposed mechanistic pathways.

In the assessment of cognitive decline in Alzheimer’s disease, determining the extent of brain tissue loss represents a critical parameter^66^. In this study, the Cavalieri principle, a stereological method, was employed to quantify hippocampal volume and to determine whether STZ-induced degeneration resulted in measurable tissue loss. The findings revealed a pronounced reduction in hippocampal volume in the ALZ group compared with all other groups. In contrast, animals treated with either curcumin or resveratrol alone exhibited preservation of hippocampal volume. However, the group receiving the combined treatment displayed a reduction in hippocampal volume comparable to that observed in the ALZ group.

The demonstration of a marked reduction in hippocampal volume following STZ administration in the present study is consistent with reports identifying hippocampal tissue loss and neuronal degeneration as core pathological features of Alzheimer’s disease models. Biasibetti et al. (2017) documented pronounced decreases in hippocampal volume and structural integrity in an STZ-induced Alzheimer model^67^, while Wartchow et al. (2025) characterized hippocampal atrophy as a defining hallmark of Alzheimer pathology in both clinical ADNI cohort data and experimental STZ models^68^. Extensive evidence from rodent studies further indicates that hippocampal shrinkage occurs rapidly, particularly within the CA1–CA3 subfields, accompanied by reductions in neuronal density, dendritic arborization, and synaptic integrity^69,70^. The substantial volume loss observed in the ALZ group in the present study aligns with this body of literature, supporting the view that STZ induces neuronal loss in the hippocampus through oxidative stress– and inflammation-mediated mechanisms.

The preservation of hippocampal volume observed with single-agent curcumin and resveratrol treatments in the present study is also in agreement with previous research. Curcumin has been shown to exert a protective effect on hippocampal pyramidal neurons by counteracting oxidative stress– and inflammation-induced degeneration, as demonstrated through both experimental and histopathological assessments^71,72^. Likewise, resveratrol has been reported to attenuate neuronal loss and tissue damage in the CA1–CA3 subfields of the hippocampus across multiple experimental models^73,74^.

The lack of synergistic efficacy observed with the combined administration of curcumin and resveratrol may be attributable to pharmacokinetic and metabolic interactions between the two polyphenols. Ge et al. (2015) reported that curcumin inhibits phase II efflux transporters such as MRP2 and BCRP, thereby restricting the export of resveratrol glucuronide and sulfate metabolites and markedly altering its intracellular bioavailability and metabolic balance^75^. Brodzicka et al. (2024) further highlighted that both curcumin and resveratrol modulate transporters including P-gp, MRP2 and BCRP, which may unpredictably reshape the pharmacokinetic profile when the compounds are co-administered^76^. In addition, curcumin is known to strongly inhibit several phase I and II metabolic enzymes, including CYP3A4, CYP1A2, UGT and SULT, whereas resveratrol interacts with multiple CYP isoforms and modulates corresponding metabolic pathways. These enzymatic interactions suggest a substantial risk of metabolic competition and pharmacokinetic interference when the two molecules are administered together^77–79^. Collectively, these mechanisms provide a plausible explanation for the antagonistic behavior that may arise when polyphenols share overlapping biotransformation pathways.

The findings of the present study are in accordance with this body of literature. Notably, although TNF-α and Caspase-3 levels were markedly reduced by single-agent treatments, their suppression was not equally evident in the combination group, indicating that the combined regimen failed to optimize anti-inflammatory and anti-apoptotic responses. A similar pattern was observed in oxidative stress markers: the reduction in MDA levels was less pronounced under combined treatment, and the enhancement of SOD activity was minimal, suggesting an absence of additive or synergistic antioxidant effects. This phenomenon aligns with reports indicating that polyphenols may exhibit pro-oxidant tendencies at high doses or when co-administered^80,81^. The modest shifts detected in the TAS/TOS balance further support the notion that curcumin–resveratrol co-administration may generate an unpredictable interaction that limits overall antioxidant capacity. Therefore, the loss of efficacy observed in the combination group is scientifically plausible when considered within the framework of transporter inhibition, enzyme modulation, metabolic competition, and dose-dependent antagonism reported in the literature. Further studies are warranted to examine these two compounds at different dose ratios, supported by time-dependent pharmacokinetic evaluations and comprehensive metabolite profiling.

Overall, these findings expand current understanding of STZ-induced neurodegeneration and provide mechanistic insight into the differential responses elicited by curcumin and resveratrol.

## Conclusion

In conclusion, the present study demonstrates that curcumin and resveratrol exert notable neuroprotective effects in an ICV-STZ–induced rat model of Alzheimer’s disease. Both compounds effectively mitigated oxidative stress, inflammatory activity, and apoptotic signaling, as evidenced by coherent biochemical (TAS, TOS, MDA, SOD, TNF-α, caspase-3), histopathological (GFAP, caspase-3), and stereological findings. Although each agent preserved neuronal integrity and hippocampal structure when administered individually, their combination failed to produce a synergistic benefit, suggesting a potential pharmacokinetic or molecular-level antagonism. These results emphasize the importance of future dose-optimization and interaction-focused studies. Overall, the favorable safety profiles and multifaceted biological actions of curcumin and resveratrol indicate that these natural compounds may serve as promising adjunctive candidates for the management of progressive neurodegenerative disorders such as Alzheimer’s disease.

## Data Availability

Raw data can be obtained from the authors upon request. All other data generated or analyzed during this study are provided in this published article and in the supplementary information files.
